# Evaluation of extremely steep residual stress gradients based on a combined approach using laboratory-scale equipment

**DOI:** 10.1107/S1600576721010335

**Published:** 2021-11-30

**Authors:** Andreas Fischer, Sebastian Degener, Alexander Liehr, Thomas Niendorf

**Affiliations:** a Institut für Werkstofftechnik (Materials Engineering), Universität Kassel, Kassel, 34125, Germany

**Keywords:** residual stress analysis, energy-dispersive diffraction, angle-dispersive diffraction, laser surface hardening

## Abstract

Surface treatments characterized by rapid heating and cooling (*e.g.* laser hardening) can induce very steep residual stress gradients in the direct vicinity of the area being treated. These gradients cannot be characterized with sufficient accuracy by means of the classical sin^2^Ψ approach applying angle-dispersive X-ray diffraction. This can be mainly attributed to limitations of the material removal method. In order to resolve residual stress gradients in these regions without affecting the residual stress equilibrium, another angle-dispersive approach, *i.e.* the universal plot method, can be used. A novel combination of the two approaches (sin^2^Ψ and universal plot) is introduced in the present work. Prevailing limits with respect to profiles as a function of depth can be overcome and, thus, high-resolution surface layer characterization is enabled. The data obtained are discussed comprehensively in comparison with results elaborated by energy-dispersive X-ray diffraction measurements.

## Abstract 2

This paper reports on high-resolution analysis of residual stress gradients for a laser-hardened surface layer using a combined angle-dispersive X-ray diffraction approach that applies the universal plot method and sin^2^Ψ evaluation.

## 1. Introduction

Laser surface hardening is a highly localized heat treatment procedure characterized by the evolution of superior compressive residual stress states in the hardened material layer in the direct vicinity of the surface. Upon adequate treatment, local fatigue strength as well as local wear resistance, *e.g.* in the case of complex steel components, can be significantly improved. The process is based on a localized phase transformation promoted by heating only the surface layer to austenitization level, followed by rapid self-quenching to eventually form martensite. Simultaneously, this local phase transformation is accompanied by the evolution of a characteristic compressive residual stress (CRS) profile (Davis, 2002 ▸; Kostov *et al.*, 2013 ▸). Depending on the applied process parameters, the required magnitude and depth penetration of the CRS profile can be adapted to the service loads (Hügel, 1992 ▸; Fischer *et al.*, 2019 ▸; Zoch & Spur, 2015 ▸).

In order to characterize the surface layer comprehensively, various characterization techniques can be used (Schajer, 2013 ▸; Withers & Bhadeshia, 2001 ▸). Thermal treatments, such as laser surface hardening and high-frequency impulse hardening (induction hardening), both characterized by extremely high heating and cooling rates, can lead to extremely steep residual stress gradients within the immediate surface region (Bleck & Moeller, 2017 ▸; Dewi *et al.*, 2019 ▸). Although the evolution of residual stresses in laser-hardened surface layers has been considered in many cases (Solina *et al.*, 1986 ▸; Yang & Na, 1989 ▸, 1991 ▸), the focus has mostly been on the determination of the CRS transition region only, *i.e.* the depth level where the CRS crosses the zero level. High-resolution evaluation of laser-induced CRS profiles up to a distance of 25 µm from the surface has not been addressed sufficiently to date. Mostly, depth measurements are realized semi-destructively by means of the widely established angle-dispersive (AD) X-ray diffraction (XRD) method using the classical sin^2^Ψ approach (Macherauch & Müller, 1961 ▸; Meixner *et al.*, 2018 ▸). Focusing on the determination of CRS profiles to depths of <25 µm, this method is often not practicable. This is because, in particular, very fine electrochemical material removal steps of ≤1 µm are prone to error and, therefore, not to be considered a reliable process. Furthermore, the sub-surface area up to a depth of 5 µm cannot be analyzed by the classical sin^2^Ψ procedure with sufficient depth resolution. The chromium radiation often used for ferrous materials is characterized by penetration depths of up to 5 µm, the actual value depending on the applied tilting angle Ψ (Spieß *et al.*, 2019 ▸). Consequently, residual stresses from layer volumes smaller than this value cannot be resolved. In this context, high-energy XRD in the energy-dispersive (ED) mode was shown to be an adequate measuring method to obtain process-related residual stress gradients within depths of a few micrometres up to 35–60 µm (in steels) without the need to remove any material, so that the residual stress equilibrium is not affected (Meixner *et al.*, 2018 ▸; Spieß *et al.*, 2019 ▸; Liehr *et al.*, 2017 ▸; Ruppersberg & Detemple, 1993 ▸; Breidenstein *et al.*, 2017 ▸). Unfortunately, the zero crossing level cannot be determined. Furthermore, the multi-wavelength method (the ED equivalent of the AD sin^2^Ψ method) provides only very few data points in the surface region <5 µm, which is not sufficient to fully capture a steep stress gradient close to the surface. Additionally, ED measurement equipment is not available in most academic and industrial laboratories. For this reason, in past decades ED studies could only be carried out externally, *e.g.* at synchrotron beamlines (BESSY – Berliner Elektronenspeicherring-Gesellschaft für Synchrotronstrahlung, Berlin, Germany; ESRF – European Synchrotron Radiation Facility, Grenoble, France) (Genzel *et al.*, 2007 ▸; Glaser *et al.*, 2018 ▸). Thus, the limited availability of this kind of research facility hindered a widespread application of the ED approach. The synchrotron beamline reported by Genzel *et al.* (2007 ▸) has been closed in recent years. Thus, remaining beamlines are even more in demand. Furthermore, technical details are a roadblock to specific use in some cases. As an example, characterization of steep CRS gradients, whose characteristic profile prevails within a depth of up to 20 µm, cannot be conducted in a straightforward manner at the beamline ID15A (ESRF, experiment M1440, energy range up to 300 keV) in reflection geometry (Glaser *et al.*, 2018 ▸).

Besides the ED methods, AD strain scanning methods with a well defined gauge volume based on measurements in transmission mode are often applied. Staron *et al.* (2013 ▸) and Kurz *et al.* (2015 ▸) showed that a great deal of experimental effort is needed to realize a local spot size down to 5 µm in transmission mode for stress measurement. In addition, a time-consuming and very accurate arrangement of the primary beam slits and sample position is mandatory for precise measurements. The use of grazing-incidence methods is also possible at synchrotron facilities, but mainly in the context of measurements of thin coating layers (Li *et al.*, 2016 ▸). However, those efforts are restricted to particular equipment and beamlines. Furthermore, data assessment is often characterized by an ‘after beam time evaluation procedure’. In consequence, further measurements are not possible, *e.g.* in cases where the experimentally determined stress gradient is characterized by imperfections or the sample has to be changed. The community has tried to close prevailing gaps recently. A new beamline (P61A, DESY – Deutsches Elektronen-Synchrotron, Hamburg, Germany) with an energy range up to 400 keV, offering the possibility of measuring the same sample in transmission and reflection mode, has started to operate. Unfortunately, details of the setup have not been reported in the literature so far.

In order to be able to determine laser-induced steep residual stress gradients to a depth <5 µm, an alternative AD approach, *i.e.* the universal plot (UP) method, can be applied. For the UP method, sample preparation is not needed and, thus, the residual stress equilibrium is not affected. Additionally, AD XRD in such fashion is characterized by very high angular resolution. This fact compensates for the poor statistics when obtaining every single UP residual stress value. At extremely steep tilting angles Ψ, residual stresses can be obtained in very fine increments. However, the residual stress profile can only be determined for a small depth, which is essentially limited by the corresponding wavelength and material properties. As detailed before, both AD XRD approaches (reflection geometry), *i.e.* sin^2^Ψ and UP, are characterized by inherent limits with regard to the assessment of CRS gradients and transition areas, respectively. Alternative AD XRD methods (transmission geometry) are almost exclusively available at synchrotron facilities. Here, excellent spatial resolution is realized by application of specific optical slit systems, eventually confining the probed volume to a defined gauge volume inside the sample (Drakopoulos *et al.*, 2015 ▸; Schell *et al.*, 2013 ▸). In this case, it is of the utmost importance that the gauge volume considered is much smaller than the prevailing stress gradient. Alternative approaches based on high-energy diffraction microscopy can even be employed to resolve stress gradients in single grains (Turner *et al.*, 2016 ▸). Unfortunately, such studies are only feasible in the case of special sample geometries. Consequently, in terms of application, these approaches are suitable neither for analysis of the CRS gradients in laser-hardened components nor for numerous application-oriented kinds of samples. Focusing on the example of laser-hardened components, a combined measuring approach (sin^2^Ψ and UP) is introduced in the present work, opening up new possibilities in materials characterization. Through a direct comparison with results obtained by ED XRD, it is evaluated how far residual stress fields in the surface to a depth of <5 µm and in large component depths of up to 2 mm can be determined reliably by using the novel combined AD XRD approach.

## 2. Experimental setup

**Figure 1:**
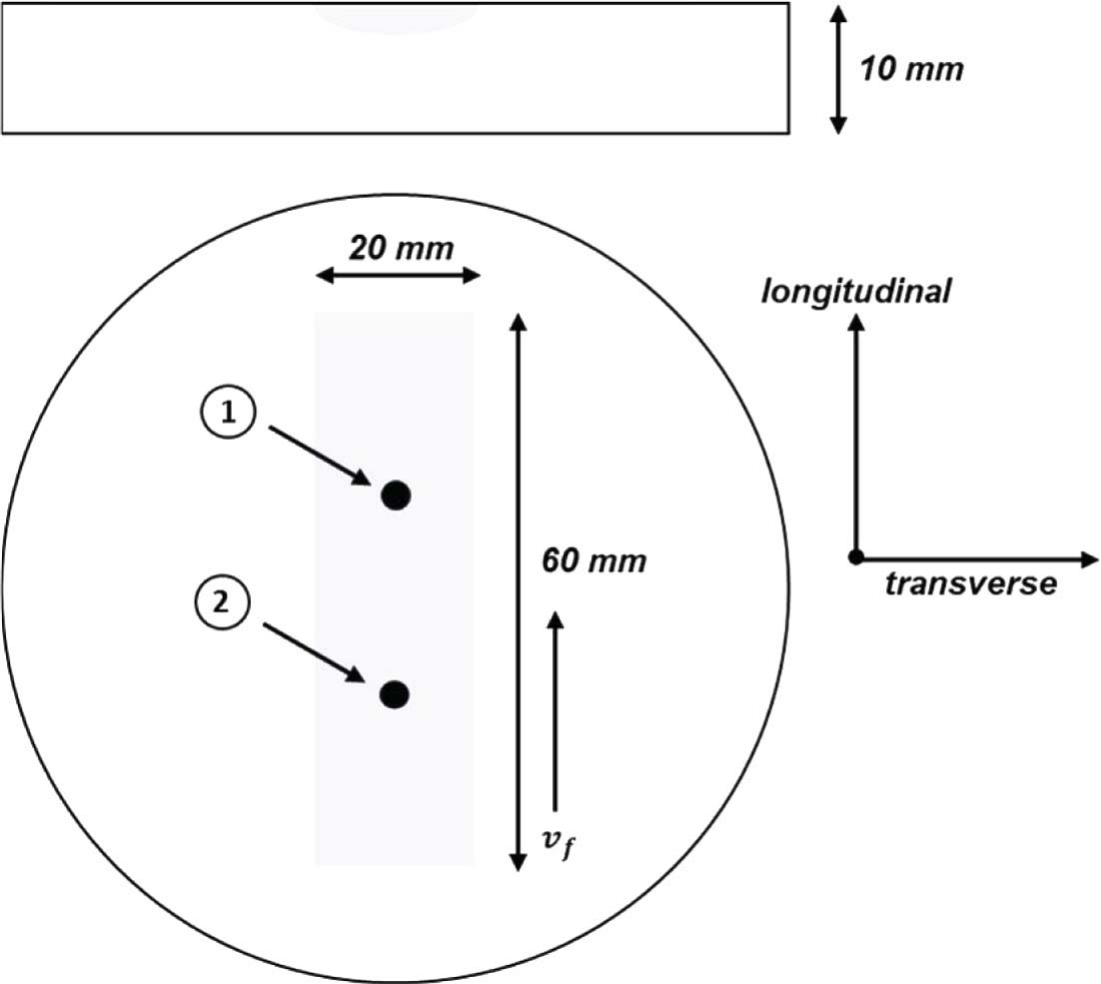
Schematic detailing the laser-hardened sample geometry, highlighting areas of interest for (1) the sin^2^Ψ method and the UP method and (2) the ED measurement.

Fig. 1 ▸ shows the dimensions of the laser-hardened specimen and the surface-hardened area (Fischer *et al.*, 2019 ▸; Dewi *et al.*, 2019 ▸). Within the laser-hardened area two measuring points were defined. At position 1 the UP method was applied first, followed by the classical sin^2^Ψ evaluation, including the corresponding material removal steps. In order to achieve a high resolution with respect to the prevailing residual stress gradient in the surface layer, very fine removal increments of around 1–2 µm down to a polishing depth of 25 µm were considered. ED XRD measurements were carried out at position 2. Because of the process-related high cooling rates occurring perpendicular to the laser feeding direction, the most pronounced residual stress gradients can be observed transversely (Fischer *et al.*, 2019 ▸). Therefore, CRS profiles were primarily investigated transversely to the laser feeding direction. Scatter values given for the applied evaluation methods were determined on the basis of ten measurements for each measuring point.

AD XRD measurements (UP and sin^2^Ψ) were performed using a four-circle diffractometer type D5000 equipped with a Cr tube source. For XRD measurements based on the classical sin^2^Ψ method, a monocapillary of 0.3 mm in diameter was applied. The diffracted intensity was measured at the (211) lattice plane of the ferrite. For each measuring point the stress evaluation was carried out using 11 equidistant Ψ angles ranging from −45 to +45°. Since CRS profiles were not used for further fatigue calculations, a layer removal correction is not necessary (Savaria *et al.*, 2015 ▸; Prevey, 1986 ▸). For this reason corrections were not done. The intensity range in the case of the UP method was measured with a step size of 0.05° at the (211) lattice plane by using a polycapillary with a diameter of 2.0 mm. In total, 18 Ψ angles in a range of 0–89° were considered with optimized counting times between 2 and 15 s.

ED XRD measurements were carried out using a diffractometer with a two-detector setup equipped with Si(Li) detectors (Liehr *et al.*, 2017 ▸; Apel *et al.*, 2018 ▸). A white beam was used consisting of the *Bremsstrahlung* generated by a conventional X-ray tungsten anode, which was operated at a voltage of 60 kV and a current of 40 mA. With a constant 2θ diffraction angle of 25° and Ψ angles tilted up to 84° (step size of 6°), the multi-wavelength method was applied to determine the residual stress depth profile σ(τ) non-destructively down to 35 µm below the surface (reflections 110 to 420 of α-ferrite).

## 3. Results

**Figure 2:**
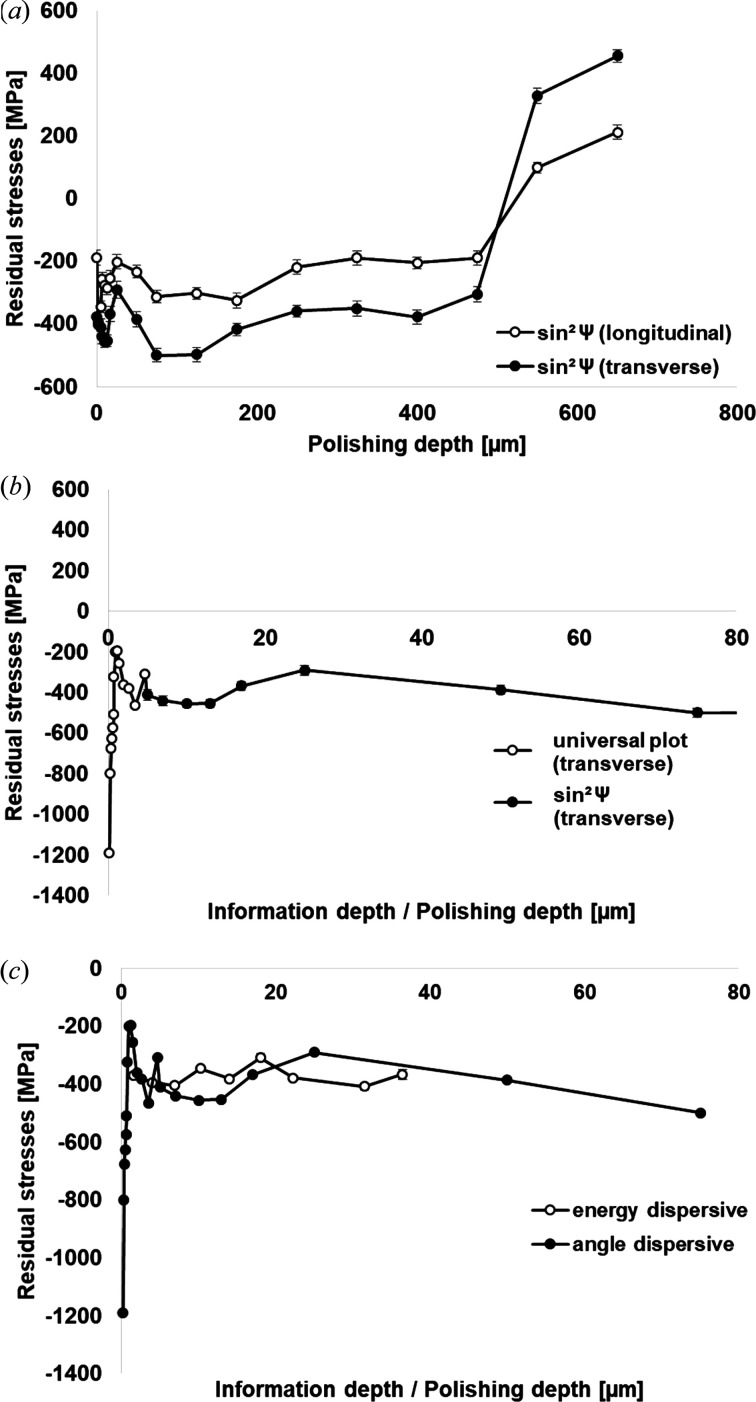
Residual stress profiles as a function of depth for a laser-hardened micro-alloyed 44MnSiVS6 steel resolved by means of (*a*) the sin^2^Ψ approach and (*b*) the combined sin^2^Ψ and UP approach; (*c*) comparison of ED and AD measurements.

Fig. 2 ▸ illustrates AD and ED data for the residual stress profile of the laser-hardened sample. According to Fig. 2 ▸(*a*) the residual stress profile in the transverse direction obtained by XRD using the classical sin^2^Ψ procedure reveals two maximum values at depths of 13 µm (−453 MPa) and 75 µm (−500 MPa). After reaching the second maximum at 75 µm, the CRS decreases slightly down to a polishing depth of 475 µm before suddenly turning into tensile residual stresses (Fischer *et al.*, 2019 ▸).

On the basis of the very fine stepwise material removal of around 1 µm down to a polishing depth of 13 µm, a very smooth increase of CRS values can be observed until the first maximum of −453 MPa (polishing depth of 13 µm) is resolved [Fig. 2 ▸(*a*)]. While the removal of such very fine increments inevitably requires enormous experimental effort, a controllable material removal of less than 1 µm and a reliable exclusion of minor relaxation and redistribution of the initial residual stress state are very challenging (Savaria *et al.*, 2012 ▸). The extremely high experimental effort of measuring residual stresses down to 5 µm can be overcome by the UP method, where material removal is not necessary. Thus, the residual stress equilibrium is not affected (Schreyer *et al.*, 2017 ▸).

Fig. 2 ▸(*b*) shows the CRS course transverse to the laser feeding direction down to a polishing depth of 75 µm in order to illustrate the process-related CRS gradient in the surface area more precisely. In this context, the evaluation based on the UP approach covers the depth range from 0.19 to 4.7 µm. Afterwards, the classical sin^2^Ψ method is used to resolve the remaining course from 5 to 75 µm. From the UP procedure a very steep and linear decreasing compressive residual stress gradient in an information depth of 0.19 µm (−1191 MPa) to 1.18 µm (−198 MPa) can be deduced. These regions cannot be determined with sufficient accuracy using the classical sin^2^Ψ method (due to preparative limits). At this point, the excellent agreement of these two methods in the transition region can be directly seen, *i.e.* both techniques reveal very similar absolute values at a depth of 5 µm. The residual stress profile obtained using the UP method shows a maximum scatter of ±42 MPa.

In order to verify the results obtained by AD XRD, the CRS state of measuring point 2 (*cf.* Fig. 1 ▸) was analyzed by means of the ED approach. Based on these data, a comparison of AD and ED measurements is presented in Fig. 2 ▸(*c*).

## 4. Discussion

**Figure 3:**
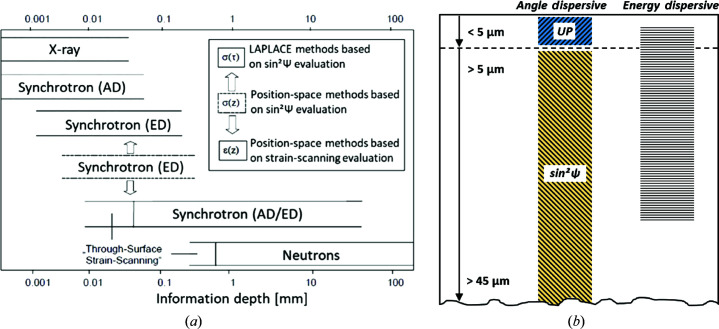
Schematics highlighting potential measuring depths (*a*) using different non-destructive diffraction methods [recompiled from the work of Denks (2008 ▸)] and (*b*) based on the AD and ED measurements using laboratory equipment only. In the case of the sin^2^ψ evaluation procedure, stepwise layer removal was realized by electropolishing.

From the literature it is known that various radiation sources and methods for data analysis can be employed for characterization of CRS states as a function of depth. Fig. 3 ▸(*a*) highlights the commonly considered approaches, where XRD is characterized by a relatively low information depth and neutron diffraction exhibits opposite characteristics. Focusing on XRD analysis using laboratory sources, the following considerations have to be taken into account: residual stress values from sample volumes below τ_0_ are accessible neither by the UP method nor by the sin^2^Ψ method in AD mode without material removal. As is highlighted in Fig. 3 ▸(*b*), ED measurements (*e.g.* the multi-wavelength method) under laboratory conditions can be applied (when appropriate systems are available) to obtain information down to a depth of around 30–50 µm in steels. The sin^2^Ψ method coupled with material removal can provide data down to depths of several millimetres. Both the sin^2^Ψ method and the multi-wavelength method are characterized by robustness due to excellent statistics (as data are averaged over a number of tilting angles). This is the reason why the sin^2^Ψ method is widely used for standardized residual stress analysis. This holds true as long as no steep stress gradients prevail inside the evaluated gauge volume. With the sin^2^Ψ method (including electrolytic layer removal), residual stresses can be determined from a few micrometres down to 1 or 2 mm under the specimen surface. The ED approach can generally address information depths of 10–70 µm with an energy range of 10–100 keV. Furthermore, the ED approach allows for completely non-destructive measurements and, concomitantly, the opportunity to adapt the related information depths by changing the diffraction angle and the selection of analyzed diffraction peaks (of different lattice planes).

An excellent agreement between AD and ED data and CRS profiles can be clearly seen in Fig. 2 ▸(*c*). ED data are characterized by scatter of only ±16 MPa. Thus, the reliability of the combined AD approach is proven, even though elaborate residual stress corrections were not done. The excellent agreement indicates that errors in terms of measurement as well as preparation can be neglected. Using the ED XRD approach, the residual stress profile could only be determined from an information depth of 1.52–36.44 µm. In comparison with the residual stress values obtained by the UP method, the process-induced steep gradient in the direct vicinity of the surface (depth of 0–1 µm) cannot be resolved by the ED approach. Focusing on the results, it is important to note that the minimum applied incidence angle of about 0.98° considered in the AD XRD measurements is 1.7 times larger than the critical angle and, thus, total reflection does not have to be considered. Any influences of mechanical preparation and additional phases in the sub-surface area on the XRD profile could be excluded by additional XRD measurements (results not shown). Therefore, the prevailing gradient can be solely attributed to a more pronounced martensitic distortion in the surface layer.

As is highlighted above, the CRS state upon laser surface hardening, characterized by a very sharp gradient directly at the surface followed by a relatively smooth gradient below (down to depths of around 100 µm), cannot be assessed by only one XRD characterization method. In general, various non-destructive diffraction methods have been proposed and established to analyze complex residual stress gradients (Denks, 2008 ▸). In the case of an extremely steep residual stress gradient, studied in the present work using laboratory equipment only, the combination of two synergetic methods is advantageous, as is illustrated by the information depth ranges shown in Fig. 3 ▸. The classical sin^2^Ψ procedure with its stepwise material removal cannot be applied in a robust and reliable fashion to analyze the first few micrometres (down to 5 µm) with a sufficient depth resolution, because the mean penetration depth of, for example, chromium radiation is between 4 and 5 µm, ultimately depending on the actual tilting angle Ψ. This intrinsic information depth is often neglected when considering material removal steps of higher magnitude. Thus, analysis of residual stress gradients that are extremely steep, *i.e.* characterized by significant changes within the minimum possible volume probed, is not feasible. In contrast, the UP method can easily capture smaller volumes due to its characteristic penetration depths being below the information depth τ_0_, eventually yielding a number of data points equal to the number of tilting angles Ψ, as highlighted in (1) (Ruppersberg *et al.*, 1989 ▸):

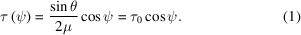

The information depth τ_0_ itself is defined by the absorption coefficient μ and by the diffraction angle θ and, thus, is constant for a specific material and investigated lattice plane. Consequently, increased tilting angles lead to a smaller information volume in the direct vicinity of the surface area. This effect is exploited by the UP method by evaluating data obtained at increased Ψ angles (>+45°), the latter being employed as a boundary limit in the case of the sin^2^Ψ procedure. As a very sharp residual stress gradient within the first 5 µm (below the surface) is characteristic for the laser-hardened condition of the micro-alloyed steel, only application of the UP method at very high tilting angles up to +89° allows for high-resolution analysis. In the approach discussed here, every tilting angle leads to one residual stress value that is representative for a distinct penetration depth, this value being explicitly defined by radiation absorption and the actual geometrical beam path.

Because only one tilting angle is analyzed for one residual stress value, the data quality crucially needs to be very high to avoid inaccuracy and scattering of the determined CRS distribution. Therefore, it is very challenging to apply the UP method to ED data experimentally determined under laboratory conditions, where the primary beam intensities and, consequently, the diffracted intensities are relatively low. In theory, information depths of less than 1 µm to several micrometres can be resolved non-destructively with the UP method.

## 5. Summary

In conclusion, it could be shown that the combined angle-dispersive XRD approach introduced in the present work paves the way towards robust and reliable surface layer characterization, allowing for assessment of high-resolution residual stress profiles (as a function of depth) in industrial and academic fields. Extremely steep CRS gradients induced by laser hardening of micro-alloyed steel in the direct vicinity of the surface (<5 µm) as well as far-reaching CRS states (*e.g.* 1 mm) can be characterized by XRD using laboratory equipment only, just considering the widely established AD mode. This opens up new research opportunities in the field of high-resolution residual stress analysis (as a function of depth). In fact, the approach introduced here is especially suitable for analysis of treated surface layers characterized by very steep residual stress gradients. The approach relies on data analysis applying the UP and the sin^2^Ψ methods. The obtained data have been critically assessed via comparison with well established ED XRD measurements. Because of the very fine depth increments characteristic for the UP approach, even a superior depth resolution in the direct vicinity of the surface can be achieved as compared with the ED XRD method. It is emphasized that strict limits prevail with respect to the component geometry in the case of the combined AD XRD approach. The accessibility of the area of interest must be ensured, which is a major drawback due to the very steep diffraction angles Ψ of up to +89° needed for data acquisition. For relatively flat component geometries the combined approach introduced here provides a flexible, robust and reliable method for residual stress analysis in most laboratory environments.
